# 12/15-Lipoxygenase Regulation of Diabetic Cognitive Dysfunction Is Determined by Interfering with Inflammation and Cell Apoptosis

**DOI:** 10.3390/ijms23168997

**Published:** 2022-08-12

**Authors:** Qi Chen, Qixue Zheng, Yang Yang, Ying Luo, Hong Wang, Huan Li, Lu Yang, Congli Hu, Jiahua Zhang, Yuke Li, Hui Xia, Zhihao Chen, Jie Ma, Xiaoyan Tian, Junqing Yang

**Affiliations:** 1 Chongqing Key Laboratory of Biochemistry and Molecular Pharmacology, Department of Pharmacology, Chongqing Medical University, Chongqing 400016, China; 2 Pharmacy Department of Guizhou Provincial People’s Hospital, Guiyang 550002, China; 3 Department of Pharmacology, Chongqing Health Center for Women and Children, Chongqing 400016, China

**Keywords:** 12/15-lipoxygenase, regulation, diabetic cognitive dysfunction, inflammation, cell apoptosis

## Abstract

This study aimed to discuss the role of 12/15-lipoxygenase (12/15-LOX) regulation involved in diabetes cognitive dysfunction. First, Mini Mental State Examination (MMSE) test was used to evaluate cognitive ability in diabetic patients and normal controls. The plasma test showed that the plasma level of 12/15-LOX in patients with MMSE scores below 27 was significantly increased compared with that of the normal group. Second, 12/15-LOX inhibitor was administered to diabetic rats. Behavioral tests, biochemistry, enzyme-linked immunosorbent assays, and Western blotting were used in this study. We found that the levels of fasting and random blood glucose increased rapidly in diabetic rats, the levels of triglycerides and total cholesterol in the diabetic group increased, and insulin levels decreased significantly. In the Morris water maze test, the escape latency was prolonged, and the crossing times decreased in the diabetic group. Under the microscope, the apoptosis of hippocampal neurons in diabetic rats increased significantly. The levels of TNF-α, IL-6 and 12-hydroxyindoleic acid (12(S)-HETE) significantly increased, and the protein expression of 12/15-LOX, p38 MAPK, Aβ_1-42_, caspase-3, caspase-9 and cPLA2 increased, while that of Bcl-2 decreased. However, the use of 12/15-LOX inhibitor reversed these results. Third, 12/15-LOX shRNA and p38MAPK inhibitor were administered to HT22 cells in high-glucose medium. The results of the cell experiment were consistent with those of the animal experiment. Our results indicated that the 12/15-LOX pathway participates in diabetic brain damage by activating p38MAPK to promote inflammation and neuronal apoptosis, and intervention 12/15-LOX can improve diabetic cognitive dysfunction.

## 1. Introduction

Diabetes is a typical chronic metabolic disease, may lead to a variety of complications, and has become the main cause of death or disability. In 2019, the International Diabetes Federation (IDF) statistics showed that there were approximately 463 million people with diabetes in the world between the ages of 20 and 79, and the number of patients with diabetes is expected to increase to 700 million in 2045 [1]. Central nervous system (CNS) disorders and cognitive dysfunction are also considered to be the most serious complications caused by diabetes [2]. Diabetes promotes central nervous system disease through a variety of mechanisms, leading to atrophy of the hippocampus and cortex in the brain and resulting in cognitive dysfunction [3]. Meyer et al. found that 17.1% of patients with cognitive dysfunction suffered from diabetes, while the prevalence of diabetes in normal cognitive function was only 4.4% [4]. As early as 1993, Akaishi T indicated that hyperglycemia directly causes brain neuron damage through experiments in vitro and in vivo [5]. However, the pathogenesis of diabetes-associated cognitive dysfunction is not well understood, and no particular method has been found for diagnosing and treating this disease. Accordingly, further studies and discussions on how diabetes leads to and exacerbates cognitive dysfunction in patients could contribute to the discovery of new drug targets and help develop novel therapies.

With the deepening of research on diabetes and cognitive dysfunction, an increasing number of studies have confirmed that inflammation plays an extremely important role in the development of diabetes. Ragy M et al. [6] and Gorska C et al. [7] found that blood levels of TNF-α and IL-6 are increased in patients with diabetic cognitive dysfunction and that the levels of these inflammatory factors are correlated with the degree of cognitive dysfunction. Animal experiments have also confirmed that IL-6 overexpression accelerates neuronal apoptosis and further leads to the progression of cognitive dysfunction [8]. Several studies have also found that inflammatory reactions play an important role in the damage induced by chronic degenerative neurological diseases such as Alzheimer’s disease (AD) and Parkinson’s disease (PD) [9,10]. The relationship between the inflammatory response and cognitive dysfunction in diabetes has gradually become the focus of recent research [11,12,13]. During the inflammatory phase, the arachidonic acid (AA) metabolic pathway is an important central link in the inflammatory response. Several key enzymes in the AA pathway produce a large number of inflammatory factors through AA metabolism. For example, both cyclooxygenase 2 (COX2) and 5-lipoxygenase (5-LOX) are important metabolic rate-limiting enzymes in the AA pathway and important inflammatory factors [14,15]. In the peripheral nervous system, activation of sirtuin 1 can improve insulin resistance, increase glucose sensing and insulin secretion and brain insulin resistance drives diabetes related cognitive decline by inhibiting sirtuin 1 signaling [16,17]. Activated sirtuin 1 can inhibit lipoxygenase and cyclooxygenase resulting in the reduction in the proinflammation pathways in the patients with cognitive impairment [18]. 12/15-lipoxygenase (12/15-LOX) is also a member of the lipoxygenase family and plays an important role in the AA metabolic pathway. 12/15-LOX and its metabolite 12-hydroxyindoleic acid (12(S)-HETE) are further involved in the development of various inflammatory diseases in the body [19,20].

12/15-LOX also participates in the study of diabetes and its complications. 12/15-LOX also participates in the study of diabetes and its complications. Type 1 diabetes is caused by absolute hyperglycemia due to insulin deficiency, while 12/15-LOX is expressed in type 1 diabetes during β Cells and macrophages were induced, and the production was exacerbated β Proinflammatory lipids and lipid peroxides for cellular dysfunction and macrophage activity. Inhibition of 12/15-LOX provides a potential therapeutic approach to prevent the deterioration of blood glucose in T1D [21]. A high-fat diet induces the increasing expression of 12/15-LOX in adipose tissue of obese rats, while 12/15-LOX inhibitors improve insulin resistance and the dysfunction of islet β and α cells in obese rats [22]. Further studies found that increasing the expression of 12/15-LOX may exacerbate the oxidative stress in murine islet β cells, leading to various diabetes complications, such as retinopathy, nephropathy, and cardiovascular disease [23,24,25,26,27]. It is found that 12/15-LOX is widely distributed in the central nervous system, mainly in the cerebellum, basal ganglia and hippocampus and is highly expressed in neurons and glial cells. Yao et al. found that the downstream metabolites of 12/15-LOX, 12(S)-HETE and 15-HETE were significantly increased in the cerebrospinal fluid of patients with AD, suggesting that 12/15-LOX and its metabolic pathways may be involved in the occurrence and development of AD [28]. Inhibition of 12/15-LOX significantly improves cognitive dysfunction, brain amyloidosis and tau pathology by stimulating autophagy in aged triple transgenic mice [29]. Some studies have also found that 12/15-LOX is increased in the brains of local or global cerebral ischemia-reperfusion injury and stroke, and inhibiting its synthesis can reduce damage [30,31,32]. 12/15-LOX also exacerbates injury in subarachnoid hemorrhage [33]. These studies suggested that 12/15-LOX may be a target for the treatment of central nervous system diseases, including diabetes-associated cognitive dysfunction. However, there are no reports in human or animal experiments. Based on these studies, we hypothesized that 12/15-LOX may be a biomarker in the diagnosis of diabetic cognitive dysfunction.

Therefore, in this study, we aimed to detect the plasma levels of 12/15-LOX in patients with diabetes-associated cognitive dysfunction and established a rat model of diabetic cognitive dysfunction to observe 12/15-LOX expression in the hippocampus. 12/15-LOX inhibitor, 12/15-LOX shRNA and p38MAPK inhibitor were used to observe the effects and explore the mechanisms of cognitive dysfunction in diabetic rats. The results of this study may provide a therapeutic target for diabetic cognitive dysfunction.

## 2. Results

### 2.1. MMSE Scores

All of them were tested following the MMSE (Table 1). The MMSE was divided into five categories: orientation, memory, attention and calculation, recall, and language ability. According to the total score determined by the MMSE, diabetic patients were divided into two groups, one with an MMSE score > 27 and the other with an MMSE score < 27. The results showed that the scores of the five aspects of diabetic patients were all decreased compared with those of the normal group, but only the attention and calculation scores showed statistically significant differences. The remaining four scores did not show significant differences. Compared with the scores of the normal group, there was no significant difference in the scores of language ability and recall ability in diabetic patients with MMSE scores < 27(*p* > 0.05). However, the orientation, memory, attention and calculation scores in diabetic patients with MMSE scores < 27 were decreased significantly compared with those of the normal group (*p* < 0.05).

### 2.2. Level of 12/15-LOX in the Plasma

**Figure 1:**
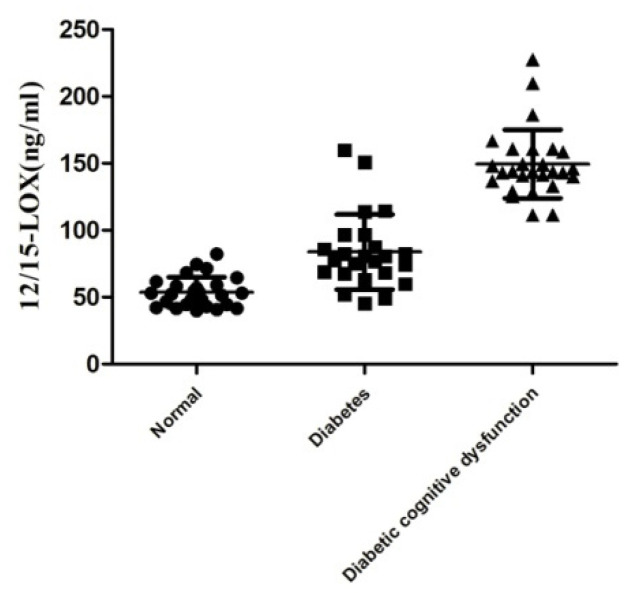
12/15-LOX is related to cognitive dysfunction in diabetes patients. The level of 12/15-LOX in the plasma of the diabetic patients with cognitive dysfunction.

Compared with the levels in the normal group (Figure 1), the plasma 12/15-LOX levels in diabetic patients with MMSE scores > 27 were significantly increased (90.12 ± 11.08 vs. 49.54 ± 2.672), and the difference was statistically significant (*p* < 0.05). The plasma 12/15-LOX levels in diabetic patients with MMSE scores < 27 points were significantly higher than (144.2 ± 4.585) those of the normal group and diabetic patients with MMSE scores < 27 (*p* < 0.01), and the difference in this group was smaller than that of the diabetes group with MMSE scores > 27.

### 2.3. Effect of 12/15-LOX on Fasting Blood Glucose and Random Blood Glucose in Diabetic Rats

**Figure 2:**
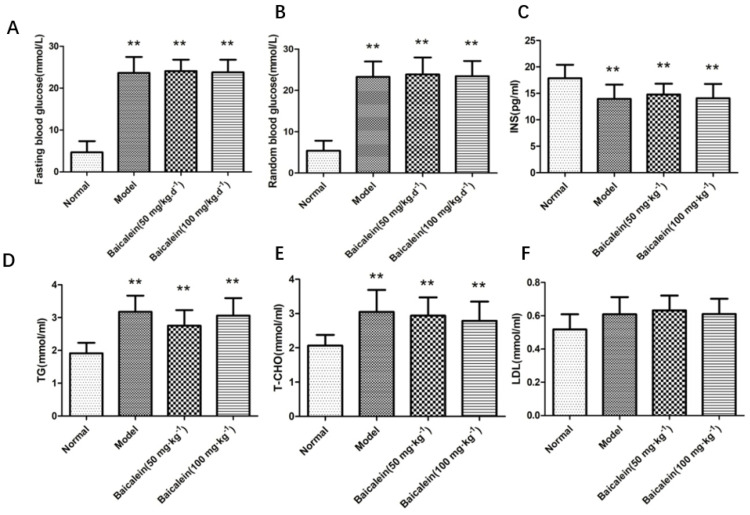
The establishment of diabetes model. Fasting blood glucose level (**A**). Random blood glucose level (**B**). Insulin level (**C**). TG level (**D**). T−CHO level (**E**). LDH level (**F**). ** *p* < 0.01 compared with normal group.

The results in Figure 2 show that fasting blood glucose and random blood glucose were significantly increased in the diabetes group compared to those of the normal group (*p* < 0.01). After the administration of 50 ^−1^ and 100 mg·kg^−1^ Baicalein, there was no significant difference in fasting blood glucose and random blood glucose in diabetic rats compared with those of the model group (*p* > 0.05).

### 2.4. Effects of 12/15-LOX on the Changes in Insulin, TG, TC and LDL-C Levels in the Plasma of Rats with Diabetic Cognitive Dysfunction

The plasma insulin level in the diabetic model group significantly decreased (Figure 2) compared with that of the normal group, and the levels of TG, TC and MDA increased significantly (*p* < 0.05, *p* < 0.5, *p* < 0.0001), but the levels of LDL did not show a statistically significant difference (*p* > 0.05). Compared with the levels in the model group, MDA level decreased after administration of Baicalein (50 and 100 mg·kg^−1^) in diabetic rats (*p* < 0.5). Compared with the model group, there were no significant differences in the levels of insulin, TG, TC and LDL after administration of Baicalein (50 and 100 mg·kg^−1^) in diabetic rats (*p* > 0.05).

### 2.5. Effects of 12/15-LOX on Changes in Learning and Memory Functions in Rats with Diabetic Cognitive Dysfunction

**Figure 3:**
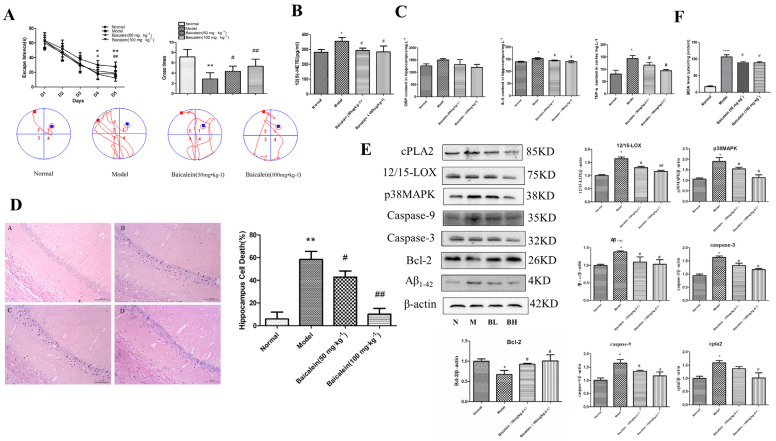
The effect and mechanism of 12/15-LOX in hippocampal injury in diabetic rats. Morris water maze test (**A**). The change in 12(S)-HETE in diabetic rats hippocampal (**B**). The change in inflammatory factor in diabetic rats hippocampal (**C**). Changes in hippocampal pathology diabetic rats (**D**). Changes in hippocampus-related proteins in diabetic rats (**E**). MDA level (**F**). * *p* < 0.05, ** *p* < 0.05 and **** *p* < 0.0001 compared with normal group; # *p* < 0.05, ## *p* < 0.01 compared with model group.

The Morris water maze results (Figure 3A) showed that rats in all groups exhibited a rapid reduction in their escape latencies to find the platform over the 4 training days. The escape latency was significantly prolonged on the 3rd and 4th days, and the cross times on the 5th day were significantly decreased in the model group compared with those of the normal groups. On the other hand, the escape latencies in the 12/15-LOX inhibitor groups (50 and 100 mg·kg^−1^ Baicalein) significantly decreased on the 3rd and 4th days, and crossing times increased on the 5th day compared with those of the model groups.

### 2.6. Effects of 12/15-LOX on the Changes in Neuronal Morphology in the Hippocampus of Rats with Diabetic Cognitive Dysfunction

In the normal group, the morphological structure of the hippocampus was intact and clear, and the model group showed marked karyopyknosis and nuclear hyperchromatism. However, the administration of Baicalein (50 and 100 mg·kg^−1^) significantly alleviated karyopyknosis in a dose-dependent manner (Figure 3D).

### 2.7. Effects of 12/15-LOX on Changes in C Reaction Protein (CRP), Tumour Necrosis Factor-α (TNF-α) and Interleukin-6 (IL-6) in the Hippocampus of Rats with Diabetic Cognitive Dysfunction

The results (Figure 3C) showed that the levels of TNF-α and IL-6 in the hippocampus of the model group were significantly higher than those in the normal group (*p* < 0.05). Although the CRP level was elevated, it was not statistically significant. Compared with levels in the model group, the levels of TNF-α and IL-6 in the hippocampus of the Baicalein group were significantly decreased in a dose-dependent manner (*p* < 0.05).

### 2.8. Changes in 12/15-LOX, p38MAPK, Aβ_1-42_, Caspase-3, Caspase-9, Bcl-2, Phospho-p38MAPK and cPLA2 Expression in the Hippocampus of Rats with Diabetic Cognitive Dysfunction

In Figure 3E, the expression levels of 12/15-LOX, p38MAPK, Aβ_1-42_ caspase-3, caspase-9, phospho-p38MAPK and cPLA2 were significantly increased in the model group compared with those in the control group, and Bcl-2 was significantly reduced in the model group. The administration of Baicalein significantly blunted the changes in 12/15-LOX, p38MAPK, Aβ_1-42_, caspase-3, caspase-9, phospho-p38MAPK and cPLA2 protein expression and increased the expression of Bcl-2 in the hippocampus of diabetic rats in a dose-dependent manner.

### 2.9. Changes in 12(S)-HETE Levels in the Hippocampus of Rats with Diabetic Cognitive Dysfunction

12(S)-HETE is an important metabolite of 12/15-LOX. Our experiments showed (Figure 3B) that the level of 12(S)-HETE in the hippocampus of the model group was significantly higher than that of the normal group (*p* < 0.05). After administration of Baicalein, the level of 12(S)-HETE in the hippocampus was significantly lower than that of the diabetic model group (*p* < 0.05).

### 2.10. Effect of 12/15-LOX on HT22 Cells Treated with High-Glucose Medium

**Figure 4:**
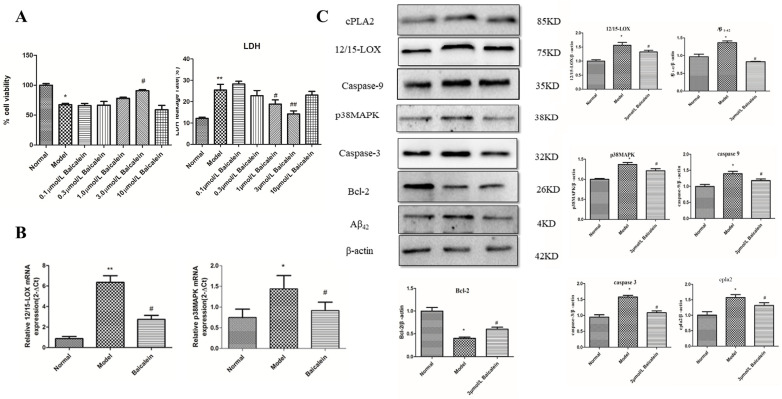
The effect and mechanism of 12/15-LOX in HT22 cells damaged by high glucose. The effect of Baicalein in HT22 cells damaged by high glucose (**A**). Changes in 12/15-LOX and p38MAPK mRNA in HT22 cells damaged by high glucose (**B**). Changes in proteins in HT22 cells damaged by high glucose (**C**). * *p* < 0.05, ** *p* < 0.05 compared with normal group; # *p* < 0.05, ## *p* < 0.01 compared with model group.

To observe the protective effect of Baicalein on HT22 cells injured by high glucose, we first detected the survival rate of HT22 cells in high-glucose medium by the MTT method. The results (Figure 4A) showed that the cell survival rate of the model group was significantly lower than that of the normal group, and the cell survival rate gradually increased with increasing drug concentrations. After administration of 3 μmol/L Baicalein, the cell survival rate was 90.69 ±1.066% compared with that of the model group (69.39 ± 1.30%) (*p* < 0.05), but the cell survival rate at the higher concentration of Baicalein (10 μmol/L) was only 58.88 ± 4.316%. Although there was no significant difference from the model group, the survival rate was significantly lower than that of the normal group, which indicated that high concentrations of Baicalein caused cell damage and reduced cell survival.

### 2.11. Effect of 12/15-LOX at Different Concentrations on LDH Leakage in HT22 Cells Treated with High Glucose

To further verify the protective effect of Baicalein on high glucose-induced HT22 cells, we used a biochemical kit to detect the leakage rate of lactate dehydrogenase (Figure 4A). Compared with the leakage rate in the normal group, the leakage rate of LDH in the model group significantly increased (*p* < 0.01). Different concentrations of Baicalein significantly reduced the leakage rate of LDH and showed dose dependence compared to those of the model group; significant effects were observed at concentrations of 1.0 and 3.0 μmol/L Baicalein (*p* < 0.05 and *p* < 0.01). However, 10 μmol/L Baicalein increased the LDH leakage rate, and there was no significant difference compared with that of the model group.

### 2.12. Changes in 12/15-LOX and p38 MAPK mRNA Expression in HT22 Cells Treated with High Glucose

In addition, the mRNA expression of 12/15-LOX (*p* < 0.01) and p38 MAPK (*p* < 0.05) increased significantly in high glucose-loaded HT22 cells compared with that of the normal group (Figure 4B). After administration of 3.0 μmol/L Baicalein, 12/15-LOX mRNA and p38 MAPK mRNA were significantly decreased compared with those of the model group (*p* < 0.05).

### 2.13. The Protein Expression of 12/15-LOX, p38 MAPK, Aβ_42_, Caspase-3, Caspase-9, Bcl-2, Phospho-p38 MAPK and cPLA2 in HT22 Cells Treated with High-Glucose Medium

Figure 4C shows the expression levels of 12/15-LOX, p38 MAPK, Aβ_1-42_, caspase-3, caspase-9, phospho-p38 MAPK and cPLA2 were significantly increased in the model group compared with those of the normal group, and Bcl-2 was significantly reduced in the model group. The administration of Baicalein (3.0 μmol/L) significantly blunted the changes in 12/15-LOX, p38MAPK, Aβ_1-42_, caspase-3, caspase-9, phospho-p38 MAPK and cPLA2 protein expression but increased the expression of Bcl-2 in HT22 cells treated with high-glucose medium.

### 2.14. Effect of 12/15-LOX shRNA Transfection on the Survival Rate of HT22 Cells Treated with High-Glucose Medium

**Figure 5:**
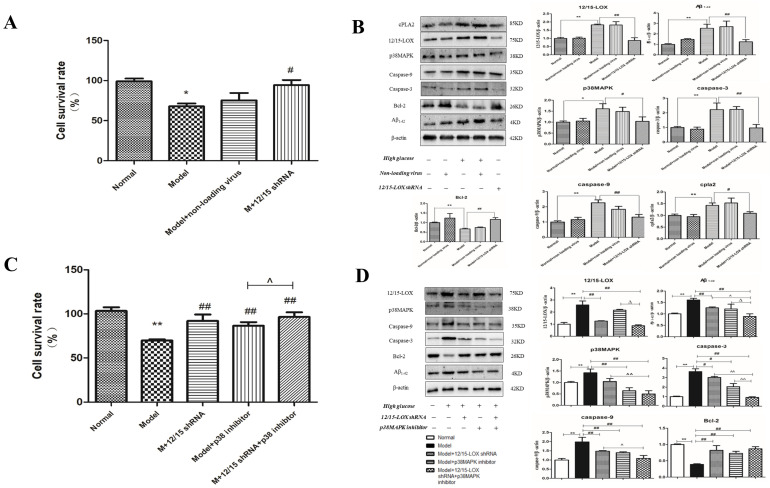
The effect and mechanism of lentiviral knockdown 12/15-LOX in HT22 cells damaged by high glucose. The effect of knockdown 12/15-LOX in HT22 cells damaged by high glucose (**A**). Changes in proteins in knockdown 12/15-LOX HT22 cells damaged by high glucose (**B**). The effect of knockdown 12/15-LOX with p38MAPK inhibitor in HT22 cells damaged by high glucose (**C**). Changes in proteins in knockdown 12/15-LOX with p38MAPK inhibitor in HT22 cells damaged by high glucose (**D**). * *p* < 0.05, ** *p* < 0.01 compared with normal group; # *p* < 0.05, ## *p* < 0.01 compared with model group. ^ *p* < 0.05 and ^^ *p* < 0.05. ∆ *p* < 0.05 and ∆∆ *p* < 0.05.

After 12/15-LOX shRNA lentivirus was stably transfected into HT22 cells, as depicted in Figure 5A, the cell survival rate of the model group was significantly reduced compared with that of the normal group (*p* < 0.05). Compared with survival in the model group, the cell survival rate of HT22 cells in the control virus group was not significantly affected by high-glucose medium, but the survival rate of 12/15-LOX shRNA transfected cells was significantly higher than that of the model group (*p* < 0.05).

### 2.15. Effect of 12/15-LOX shRNA on the Expression of 12/15-LOX, p38 MAPK, Aβ, Caspase-3, Caspase-9, Bcl-2, Phospho-p38 MAPK and cPLA2 Protein in HT22 Cells Treated with High Glucose

The expression of 12/15-LOX, p38MAPK, caspase-3, caspase-9, cPLA2, phospho-p38 MAPK and Aβ was significantly increased (Figure 5B) in the model group compared with that of the normal group (*p* < 0.01 and *p* < 0.05), and the control virus did not significantly affect the expression of these proteins (*p* > 0.05). The expression of 12/15-LOX, p38MAPK, Aβ, caspase-3, caspase-9, phospho-p38MAPK and cPLA2 was significantly decreased compared with that of the model group (*p* < 0.01 and *p* < 0.05), but the expression of Bcl-2 showed the opposite effect (*p* < 0.01) after the transfection of 12/15-LOX shRNA (*p* > 0.05).

### 2.16. Effect of 12/15-LOX shRNA and p38MAPK Inhibitor on the Cell Survival Rate of HT22 Cells Treated with High Glucose

The cell survival rate of the model group was significantly reduced (Figure 5C) compared with that of the normal group (*p* < 0.05). The cell viability in the 12/15-LOX shRNA transfection, p38MAPK inhibitor and dual intervention groups all increased significantly compared with that of the model group (*p* < 0.01). Moreover, the cell survival rate of the double intervention group was significantly higher than that of the p38MAPK inhibitor group (*p* < 0.05) and higher than that of the 12/15-LOX shRNA transfection group, although there was no statistical significance (*p* > 0.05).

### 2.17. Effect of 12/15-LOX shRNA and p38MAPK Inhibitor on the Expression of 12/15-LOX, p38MAPK, Aβ_1-42_, Caspase-3, Caspase-9, Bcl-2 and Phospho-p38MAPK Protein in HT22 Cells Treated with High Glucose

Compared with expression in the model group, the results (Figure 5D) show the expression of p38MAPK, caspase-3, caspase-9 and phospho-p38MAPK was significantly decreased in HT22 cells treated with high-glucose medium, but Bcl-2 was significantly increased (*p* < 0.01 and *p* < 0.05), and there was no significant difference in the expression of 12/15-LOX or Aβ_1-42_ (*p* > 0.05). After dual treatment with 12/15-LOX shRNA transfection and p38 MAPK inhibitors, the protein expression of 12/15-LOX, p38MAPK, caspase-3, caspase-9, phospho-p38 MAPK and Aβ_1-42_ in HT22 cells in high-glucose medium was significantly decreased, while the expression of Bcl-2 was significantly increased. (*p* < 0.01).

Compared with expression in the 12/15-LOX shRNA transfection group, the expression levels of p38MAPK, caspase-3, caspase-9, phospho-p38MAPK and Aβ_1-42_ were significantly decreased in the dual intervention group (*p* < 0.01 and *p* < 0.05), but the expression levels of 12/15-LOX and Bcl-2 were not significantly different. Compared with expression in the p38MAPK inhibitor group, the expression of 12/15-LOX, Aβ_1-42_, phospho-p38MAPK and caspase-3 in the dual intervention group was significantly decreased (*p* < 0.01 and *p* < 0.05), while the expression of p38MAPK and caspase-9 was decreased. However, there was no significant difference in Bcl-2 expression (*p* > 0.05).

## 3. Discussion

Recently, due to the changes in lifestyle worldwide, the incidence of diabetes has increased rapidly, and diabetes as an independent risk factor for cognitive dysfunction has been proved. Additionally, the number of cases of cognitive dysfunction combined with diabetes continue to rise, and treatment is becoming a significant problem. The relationship between inflammation and diabetes has been studied for years, and the inflammatory factors that affect T2DM have been mentioned by different researchers. Some scholars even consider diabetes to be a kind of chronic inflammatory disease. Studies have shown that CNS inflammation and oxidative stress are common characteristics of acute or chronic brain injuries. Therefore, finding the key link or a new target between inflammation and diabetes may be effective in preventing or treating diabetic brain damage. 12/15-LOX is an important enzyme of AA metabolic pathway that leads to inflammation [19,20]. Some studies have indicated that activation of the 12/15-LOX accompanies metabolic decline in db/db prediabetic mice [34]. The expression of 12/15-LOX is an important part of our research and was increased in diabetic patients with cognitive dysfunction. We used the MMSE scores to measure cognitive function in diabetes patients and normal individuals. Among 41 diabetic patients, 27 patients showed low MMSE scores, and 24 patients had MMSE scores higher than 27, which was close to that of the normal group. At the same time, we found in this test that diabetic patients with lower MMSE scores mainly scored lower in orientation, memory, attention and calculation ability without any significant differences in age, gender or education level of each group. Therefore, after measuring the MMSE score, we determined 12/15-LOX levels in the plasma of diabetic patients and normal individuals. The results showed that 12/15-LOX levels in diabetic patients were higher than those in the normal group. This elevation was observed in diabetic patients with MMSE scores less than 27, and the plasma level of 12/15-LOX was higher than that of patients with diabetes who had a higher MMSE score and the normal group. These results indicate that high levels of 12/15-LOX may be involved in the pathogenesis of diabetes and diabetes-induced cognitive dysfunction.

Hyperglycemia has been reported to contribute to cognitive dysfunction through several potential mechanisms, including activation of the polyol pathway, diacylglycerol-mediated activation of protein kinase C, increased formation of advanced glycation end products (AGEs) and increased glucose shunting in the hexosamine pathway [35]. Several studies have shown that dysfunctional insulin secretion causes cognitive dysfunction [36]. Based on these studies, we also verified the mechanism by which 12/15-LOX activation leads to an increased inflammatory response and causes diabetic brain damage in animal and cell models. Our results showed that plasma insulin levels were significantly decreased, blood glucose and TC and TG levels were significantly increased in model rats, and learning and memory functions were significantly decreased. The levels of IL-6, TNF-α and 12(S)-HETE were significantly increased in the model rat hippocampus. The expression of cPLA2, Aβ_1-42_, p38MAPK, phospho-p38MAPK, caspase-3 and caspase-9 was significantly increased, while the expression of Bcl-2 was significantly decreased in the model rat hippocampus. The 12/15-LOX inhibitor did not influence insulin, blood glucose, LDL, TC or TG levels in plasma. The 12/15-LOX inhibitor significantly decreased the levels of IL-6, TNF-α and 12(S)-HETE and the expression of cPLA2, Aβ_1-42_, p38 MAPK, phospho-p38MAPK, caspase-3 and caspase-9 while significantly increasing the expression of Bcl-2. These results indicate that the 12/15-LOX inhibitor showed a significant protective effect on diabetic cognitive dysfunction mainly by inhibiting inflammation and neuronal apoptosis. Or results are similar to those of other studies: the increase in 12/15-LOX leads to widespread brain injury following global cerebral ischemia [31]. In addition, inhibition of 12/15-LOX significantly improved the cognitive deficits and neuropathological changes induced by permanent occlusion of the bilateral common carotid arteries [37]. Pallast et al. found that when 12/15-LOX is activated, it can lead to the translocation of apoptosis-inducing factor (AIF) to the neuronal nucleus, promoting the condensation of nuclear chromatin, translocation of AIF and generation of cell death [38]. The inhibition of 12/15-LOX significantly improves stroke in permanent and tPA-induced thrombolysis models [32], and taurine protects neurons through inhibition of the 12/15-LOX pathway in cerebral ischemia in rats [39]. Some studies have found that 12(S)-HETE, a 12(15)-LOX metabolite, further exacerbates cell injury by promoting the production of inflammatory cytokines through p38MAPK during the inflammatory reaction. The 12/15-LOX inhibitor downregulates the expression of p38MAPK to improve cell damage caused by diabetic peripheral neuropathy [40]. Other studies have found that 12(S)-HETE, as a product of 12/15-LOX, directly activates p38MAPK to promote inflammation and cell apoptosis [41]. These results suggested that the mechanism by which diabetes induces cognitive dysfunction is closely related to inflammation and neuronal apoptosis caused by elevated 12/15-LOX expression.

Studies have confirmed that diabetes is a main risk factor for developing AD. Increased Aβ may be a direct mechanism by which diabetes causes central nervous system injury and the progression of AD. Our experimental results also showed that the expression of Aβ was significantly increased in model rat brains and that the 12/15-LOX inhibitor significantly decreased Aβ expression. Similar to our results, it was reported that the Aβ_1-42_ level was significantly increased in the brains of T2DM patients [42], and the levels of Aβ_1-42_ and its precursor were significantly increased in the forebrain cortex of T2DM rats [43]. Some studies also found that 12/15-LOX inhibition reverses cognitive dysfunction, brain amyloidosis, and tau pathology in aged triple transgenic mice [29]. Inflammatory factors activate microglia in the brain, and activated microglia secrete more inflammatory factors, which amplify inflammation in the brain to produce a large amount of APP, which is a source of more Aβ [44]. Although we did not find the deposition of Aβ in our study, recent studies have shown that soluble Aβ is an important source of neurotoxicity and is more toxic than fibrous Aβ [45]. Some studies have shown that soluble Aβ isolated from the brain tissue of AD patients could induce hyperphosphorylation of tau protein in hippocampal neurons and damage the cytoskeleton of microtubules [46]. Some studies also found that Aβ activates microglia to release inflammatory factors to induce neuronal damage [47,48,49]. Aβ and the activation of inflammation form a vicious cycle. At the same time, our experimental results also found that the expression of cPLA2 was significantly increased. Phosphorylated p38MAPK directly induces the activation of cPLA2 to synthesize more AA and ultimately form an inflammatory cycle [50]. Therefore, the inhibition of 12/15-LOX directly inhibits not only p38 MAPK to decrease inflammation and apoptosis but also the vicious cycles of cPLA2 and inflammation, as well as Aβ and inflammation.

Hippocampal neurons are also ideal models for the study of cognitive disorders [51]. HT22 is a mouse hippocampal neuronal cell line [52]. HT22 is a good cell line for studying neuronal inflammation and apoptosis in vitro, and it has been successfully applied to in vitro models of various neurodegenerative diseases, such as Alzheimer’s disease [53] and Parkinson’s disease [54,55]. Similar to the results of other in vitro studies, we found that the 12/15-LOX inhibitor significantly increased the survival rate of high glucose-loaded HT22 cells and the rate of LDH leakage. At the same time, the 12/15-LOX inhibitor significantly decreased the expression of 12/15-LOX, Aβ_1-42_, p38 MAPK, caspase-3, caspase-9, and cPLA2 and increased the expression of the antiapoptotic protein Bcl-2 in high glucose-loaded HT22 cells.

To eliminate other possible interference, 12/15-LOX shRNA was used to knock down the expression of 12/15-LOX. 12/15-LOX shRNA transfection increased the survival rate of high glucose-loaded HT22 cells, reduced the expression of 12/15-LOX, p38 MAPK and Aβ and regulated the expression of caspase-3, caspase-9 and Bcl-2. In combination with 12/15-LOX shRNA and p38MAPK inhibitors, downstream apoptotic genes were found to be regulated by the expression of 12/15-LOX and p38 MAPK. 12/15-LOX shRNA transfection reduced p38MAPK expression, but the p38 MAPK inhibitor alone did not affect the expression of 12/15-LOX. Dual intervention with 12/15-LOX shRNA and the p38MAPK inhibitor significantly reduced the expression of Aβ, caspase-3 and caspase-9 and increased the expression of Bcl-2. These results suggest that 12/15-LOX is involved in the mechanism by which high glucose damages HT22 cells, which may be achieved by regulating the p38 MAPK pathway and thereby affecting apoptosis.

One limitation of this study is the use of only male rats. The use of male rats is to exclude the effects of gender differences, and the use of both male and female rats will make the sample standard deviation large. At the same time, male rats eat more food than female rats, and insulin resistance is easy to be modeled after high sugar and high-fat diet. Female rats have a greater impact on the experiment due to hormone effects. Furthermore, the mechanism of diabetes in female rats may be different from that in male rats; future studies will focus on addressing sex-based variations in this novel type 2 diabetic animal model.

**Figure 6:**
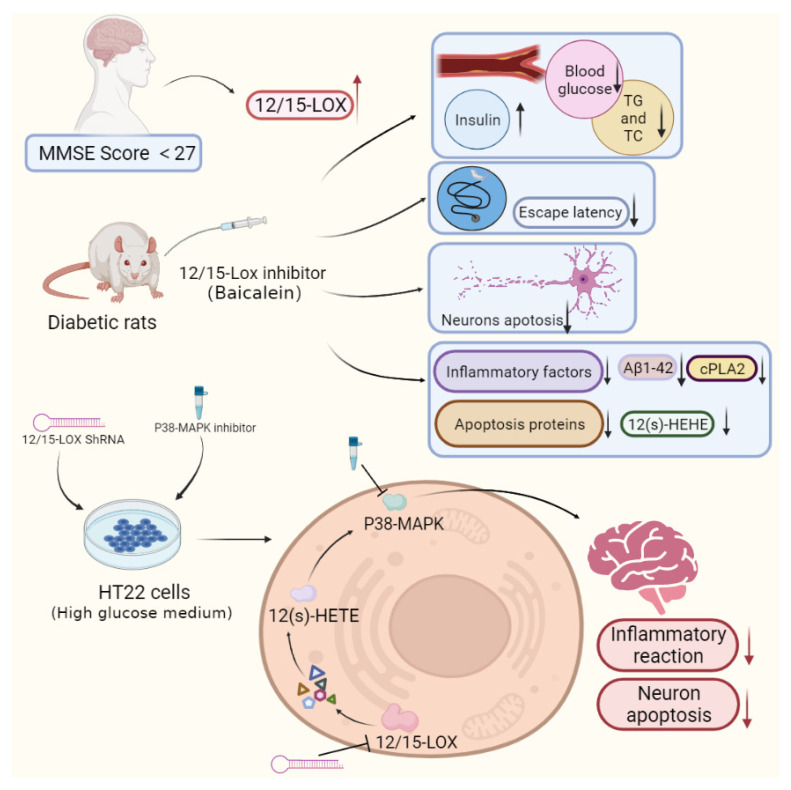
Activation of 12/15-LOX leads to diabetic cognitive dysfunction by increasing the inflammatory response in the hippocampus. 12/15-LOX regulates p38 MAPK to mediate the inflammatory response and apoptosis which aggravate diabetic cognitive dysfunction.

Based on our experimental results, increased plasma levels of 12/15-LOX were observed in patients with diabetic cognitive dysfunction. In addition, we demonstrated that activation of 12/15-LOX leads to diabetic cognitive dysfunction by increasing the inflammatory response in the hippocampus. Inhibition of 12/15-LOX reduced inflammation and damage to hippocampal neurons, downregulated the expression of p38 MAPK and phospho-p38 MAPK, decreased the production of Aβ_1-42_, and thus significantly improved cognitive dysfunction in diabetes (Figure 6). Therefore, the pathway by which 12/15-LOX regulates p38 MAPK to mediate the inflammatory response and apoptosis may be one of the mechanisms for diabetic cognitive dysfunction. We believe that 12/15-LOX may be used as a biomarker to detect diabetic cognitive dysfunction, and diabetic cognitive dysfunction can be treated or alleviated by inhibiting 12/15-LOX.

## 4. Materials and Methods

The present experiment was divided into three parts. The first part focused on testing 12/15-LOX level in plasma and MMSE scores of diabetic patients, to find out the relationship between the 12/15-LOX level in plasma and diabetic cognitive dysfunction. The second part aimed to set up the rat model of diabetes, observe the changes in behavior, blood glucose, biochemical signals, inflammation cytotoxins, 12/15-LOX and its downstream protein expression in the hippocampus of diabetic cognitive dysfunction rats after being subjected to 12/15-LOX inhibitor (Baicalein). In the third part, 12/15-LOX inhibitor, 12/15-LOX shRNA and p38MAPK inhibitor were transduced to illustrate the changes in 12/15-LOX and its downstream p38MAPK signaling pathway in HT22 cell stimulated by high-glucose medium.

### 4.1. Patients

Diabetic patients from Guizhou Provincial People’s Hospital during April 2017 to December 2018 were enrolled: aged 60 years ± 5 years old, male or female, primary education and above; the inclusion and exclusion criteria were established according to WHO diabetes diagnostic criteria in 2016. The exclusion criteria of the study subjects are as follows: (1) patients suffering from other endocrine system diseases; (2) patients with other neurological diseases, vascular diseases, craniocerebral trauma, severe visual dysfunction, hearing dysfunction, and physical activity in patients with other cognitive dysfunctions; (3) patients who are taking other drugs that affect cognitive function. A total of 51 patients with type 2 diabetes and 26 normal person were enrolled. The age, sex and fasting blood glucose of the subjects were counted. The results showed that 26 cases met the normal inclusion criteria, including 14 males and 12 females, aged 57.88 ± 2.97 years old, fasting blood glucose 4.80 ± 0.36 mmol/L; 51 cases follow the diabetes inclusion group criteria, including 28 males and 23 females, aged 61.24 ± 3.89 years old, fasting blood glucose 10.48 ± 3.24 mmol/L. This study was approved by the Ethics Committee of Guizhou Provincial People’s Hospital (Number: 2015207).

### 4.2. The MMSE 

The general clinical data of patients were collected: gender, age, education level, and diabetes history of the participants. According to “Guidelines for the diagnosis and treatment of dementia and cognitive disorders in China: Mild cognitive function Diagnosis and Treatment of Disorders” [16], using the Mini Mental State Examination (MMSE) to test and record patients’ responses, then calculate its scores. 

### 4.3. Enzyme-Linked Immunosorbent Assay (ELISA)

Plasma of the diabetic patients and the normal people were collected, plasma levels of 12/15-LOX were measured by ELISA kits (Cusabio, Wuhan, China) according to the manufacturer’s recommendation. 

### 4.4. Animal

Sprague Dawley (SD) rats were housed in the barrier housing facility, with the national standard “Laboratory Animal-Requiremtanents of Environment and Housing Facilities”. The care of laboratory animal and the animal experimental operation have conforming to “Chongqing Administration Rule of Laboratory Animal”. The experimental procedures were approved by the animal laboratory administrative center and the institutional ethics committee of Chongqing Medical University (License number: SYXK YU 2012-0001), also in accordance with the National Institutes of Health guidelines. The rats were kept in controlled conditions of temperature (24 ± 2 °C), relative humidity (60 ± 10%) and 12/12 h light/dark cycle (light from 08:00 am to 08:00 pm).

### 4.5. Establishment of Animal Models

A total of 50 male rats (80–100 g) were fed a high-fat diet (HFD) after a week of being fed a normal diet. After 4 weeks on the HFD, rats were fasted with water for 6 h and injected once with low-dose streptozotocin (Solarbio, Beijing, China) (STZ, 40 mg·kg^−1^ i.p) to induce partial insulin deficiency. Next, rats were continuously fed an HFD for 4 additional weeks after the injection of STZ. Then, the rats were fasted for 8 h, fasting blood glucose of tail vein was measured after 4 weeks of the high-fat and high-sugar diet, and a fasting blood glucose > 7.0 mmol/L was regarded as a successful establishment of the model. A total of 30 rats with diabetes were randomly and equally divided into the following 3 groups: the model group, the low-dose Baicalein group (50 mg·kg^−1^), and the high-dose Baicalein group (100 mg·kg^−1^). At the same time, 10 normal rats of the same age were randomly selected as the normal group. Rats in the Baicalein groups were subjected to intragastric administration of 12/15-LOX inhibitor (Baicalein) at a dose of 50 or 100 mg·kg^−1^ each day for 8 weeks, and the normal group and the model group were given 0.5% CMC-Na in parallel. During the eight-week feeding, 6 rats died in total, including 2 rats in the model group, 2 rats in the Baicalein (50 mg·kg^−1^) group and 2 rats in the Baicalein (100 mg·kg^−1^) group. The cause of animal death was not clear. No obvious abnormalities in the surrounding tissues and organs were detected in the autopsies of the dead rats. Therefore, there were 8 rats remaining in each group when the administration was completed. The total duration of the feeding was 14 weeks. After the end of feeding, the actual age of the rats was approximately 18 weeks.
HFDSugar20%Lard10%Egg yolk10%Basal (protein 20.6%, fat 12.0%, carbohydrate 67.4%)60%

### 4.6. Fasting Blood Glucose and Random Blood Glucose Test

At the end of feeding, rats were measured random blood glucose at any time. Fasting blood glucose were measured after the prohibition rats fasting for 6 h.

### 4.7. The Morris Water Maze Test

The Morris water maze test was used to evaluate learning and memory function of rats in each group. Rats were given four trials per day for four consecutive days. A different entry site was used for each trail. During each trial, each rat was drawn into the water where a hidden platform was submerged under it. If the rat failed to locate the platform within 90 s, it was gently guided to the platform and allowed to remain for 10 s on top of the platform, repeating the same procedure for the last three trails. On the 5th day, rats were drawn into the pool from the entry site where the last training was performed. During this trial, the platform was removed from the maze. The escape latency to find the hidden platform and the number of times crossing the platform were recorded, with a maximum of 90 s.

### 4.8. Biochemical Assays

Plasma levels of insulin (INS) was measured by ELISA kits (YuanYe, Shanghai, China) according to the manufacturer’s recommendation. The levels of triglycerides (TG), total cholesterol (TC) and low-density lipoprotein (LDL) in blood samples were measured by commercial assay kits (JianCheng, Nanjing, China*)* according to the manufacturer’s directions.

### 4.9. Enzyme-Linked Immunosorbent Assay (ELISA)

Rat hippocampus of each group was removed on the second day after the completion of Morris water maze tests (n = 4). An amount of 20 mg of rat hippocampus was added to 0.2 mL of saline for homogenate and was centrifugated at 12,000× *g* for 15 min at 4 °C, then the supernatant was used for ELISA. ELISA kits were used to detect the levels of 12(S)-HETE (Abcam, USA), CRP (YuanYe, Sahnghai, China), TNF-α (YuanYe, Shanghai, China) and IL-6(YuanYe, Shanghai, China), INS (YuanYe, Shanghai, China) 

### 4.10. Histopathological Observation

On the second day after the Morris water maze test, 4 rats from each group were chosen for histopathological observation. The rats were anesthetized and transcardially perfused with heparinised saline (100 mL) followed by 4% paraformaldehyde in phosphate-buffered saline (200 mL). The rat brain was removed and stored in the same fixative solution. The hippocampus tissue was sliced into 5 μm-thick sections for hematoxylin and eosin staining (H.E). Morphologic changes in hippocampus neurons were examined using light microscopy. High power fields were sampled from the hippocampus CA1 subfield. Cells with a distinct nucleus and nucleolus were regarded as intact neurons.

### 4.11. Western Blotting

For the rat’s hippocampus, 20 mg of rat hippocampus (n = 4) was added to 0.2 ml of tissue lysate solution for protein extraction and was centrifugated at 12,000× *g* for 15 min at 4 °C, then the supernatant was used for Western-blotting. Meanwhile, for HT22 cell, after treatment, cells were washed twice with ice-cold PBS and 200 μL lysate solution was used to lysate HT22 cells for 15 min at 4 °C, then the whole cell lysates were used for Western-blotting. The protein concentrations were determined with the BCA protein assay kit (Beyotime, Shanghai, China). A volume of 8 μL sample of each protein was separated by sodium dodecyl sulphate polyacrylamide gel electrophoresis (SDS-PAGE) and was transferred to polyvinylidene fluoride (PVDF) membranes (Millipore, Burlington, MA, USA). The membranes were blocked with 5% BSA for 4 h at room temperature and then were probed with specific primary antibodies, including 12/15-LOX(dilution 1:500; Abcam, Cambridge, UK), p38MAPK(dilution1:500; Abcam, Cambridge, UK), Aβ (dilution 1:500; Abcam, Cambridge, UK), caspase-3 (dilution 1:1000; Proteintech, Chicago, IL, USA), caspase-9 (dilution 1:1000; Proteintech, Chicago, IL, USA), BCL-2 (dilution 1:1000; Proteintech, Chicago, IL, USA), cPLA2 (dilution 1:1000; Boster, Wuhan, China) and β-actin (dilution 1:3000; Proteintech, Chicago, IL, USA) overnight at 4 °C. The membranes were washed three times in TBST and incubated with HRP-conjugated secondary antibodies at room temperature for 2 h. Following four washes in TBST, protein signals were visualized by ECL (Bio-Rad, Hercules, CA, USA).

### 4.12. Cell Culture

HT22 cells were cultured in DMEM media supplemented with 10% FBS, 100 U/mL penicillin and 100 μg/mL streptomycin in a humidified 5% (*v*/*v*) CO_2_ incubator at 37 °C. For the experiments, after cell batch and adherence for 4 h, sucked out the normal medium and then the cells were treated in 75 mM glucose medium, 3 μM Baicalein medium and 3 μM p38MAPK inhibitor medium.

### 4.13. Lentivirus Transfection

A15-LOX shRNA and non-loading lentivirus (con) were purchased from Genechem Co., Ltd. (Shanghai, China). HT22 cell stably expressing the A15-LOX shRNA targeting the sequence 5′-TTGGATAAGGAAATTGAGATT-3′ One day before transfection, 5 × 10^4^ cells per well (reaching approximately 30% confluency at the time of transfection) were cultured in T_25_ culture flask. These lentiviruses were introduced into HT22 cells treated with 10 μg/mL polybrene (Genechem, Shanghai, China) and complete medium. Transfection effects were observed by a fluorescence microscope after 96 h.

### 4.14. MTT Assay

Cell viability was determined as described based on conversion of MTT to MTT-formazan by mitochondrial enzymes as follows. Briefly, cells were seeded into a 96-well plate at a density of 1 × 10^5^ cells/well in growth medium and cultured to approximately 60–70% confluency, prior to the initiation of experimental treatment. After cell batch and adherence for 4 h, cells were treated with 3 μM Baicalein in 75 mM glucose DMEM. After 36 h of incubation at 37 °C, cells were washed three times with PBS and 20 μL of MTT solution (5 mg/mL stock) was added to the cells, and they were then incubated for 1 h at 37 °C. The medium was removed carefully and 200 μL dimethylsulfoxide was then added to resolve the blue formazan in living cells in 5 min. Finally, the absorbance at 540 nm was read with an ELISA reader (Multiskan EX, Thermo Lab Systems, Beverly, MA, USA).

### 4.15. LDH leakage 

Briefly, cells were seeded into a 96-well plate at a density of 1 × 10^5^ cells/well in growth medium and cultured to approximately 60–70% confluency, prior to the initiation of experimental treatment. After cell batch and adherence for 4 h, cells were treated with 3 μM Baicalein in 75 mM glucose DMEM. After 36 h of incubation at 37 °C the medium containing the detached cells was collected and centrifuged. The supernatant was used for the assay of LDH activity under the protocol of LDH kits *(*JianCheng, Nanjing, China*)*. A spectrophotometer was used to measure the optical density (OD) value at 490 nm.

### 4.16. Quantitative Real-Time PCR (qPCR)

Total RNA was extracted from the HT22 cells using RNAiso Plus (Total RNA Extraction Reagent, TaKaRa Bio Inc, Tokyo, Japan) in accordance with the manufacturer’s protocol. Total RNA of each group was extracted at optimal effect of induction. Approximately 1000 ng of total RNA was reverse transcribed using the Prime Script1TM RT Reagent Kit with gDNA Erase (TaKaRa Bio Inc., Tokyo, Japan). qPCR was performed in triplicate in a 20 μL volume, using SYBR1Premix Ex TaqTM14II (TaKaRa Bio Inc., Tokyo, Japan) and the CFX96 Touch TM Real-Time PCR Detection System (Bio-Rad, Hercules, CA, USA) according to the manufacturers’ instructions. Gene expression was determined relative to the housekeeping gene GAPDH using the 2^−ΔΔCt^ method. Specific primers were list as follows within (Sangon, Shanghai, China) designed. The primer sequences for 12/15-LOX were: forward 5′CATCTTCTGAGGGGACACTT3′ and reverse 5′ AGGCTCCAGCTTGCTTGAG3′, p38MAPK with forward 5′GAAGATGCTCGTTTTGGACTCAG 3′ and reverse 5′TTCAAAGGACTGGTCATAAGGGT 3′, GAPDH with forward 5′ ACAGCAACAGGGTGGTGGAC3′ and reverse 5′ TTTGAGGGTGCAGCGAACTT 3′.

### 4.17. Chemicals

Baicalein (Beyotime, Shanghai, China) was prepared to make the suspensions liquid of 50 and 100 mg·kg^−1^ with 0.5% sodium carboxy methyl cellulose (CMC-Na) (National Chemical Reagent, Beijing, China) for the rats, and 3 μM solved in 75 mM glucose DMEM was prepared for cell culture. Glucose (Kelong, Chengdu, China) was added into DMEM for preparing 75 mM medium. Efficiency of 12/15-LOX gene silencing lentivirus and no-loading virus (Genechem, Shanghai, China) transfection were determined before experiments. p38MAPK inhibitor SB 203050(Abcam, Cambridge, UK ).

### 4.18. Statistical Analysis

Data are presented as the mean ± standard deviation (SD). Statistical analysis was carried out using SPSS 20.0 statistics software (NYC, USA) and data were analyzed by performing one-way analysis of variance (ANOVA) followed by post hoc Tukey’s test. A *p* value less than 0.05 was considered statistically significant.

## Figures and Tables

**Table 1:** MMSE scores of the diabetic patients and the normal people.

Item	Normal (n = 26)	Diabetic Patients MMSE > 27(n = 24)	Diabetic Patients MMSE < 27 (n = 27)
Orientation	9.81 ± 0.39	9.67 ± 0.48	9.19 ± 0.57 *
Memory	2.75 ± 0.44	2.67 ± 0.48	2.15 ± 0.47 *
Calculation and attention	4.54 ± 0.51	4.00 ± 0.51 *	3.33 ± 0.55 ** ^#^
Recall	2.69 ± 0.47	2.42 ± 0.50	2.15 ± 0.46
Language	9.42 ± 0.50	9.04 ± 0.46	8.70 ± 0.67

* *p* < 0.05, ** *p* < 0.01, ^#^ *p* < 0.05.

## Data Availability

The data that support the present results are available from the corresponding author upon reasonable request.
